# Higher Dietary Inflammation in Patients with Schizophrenia: A Case-Control Study in Korea

**DOI:** 10.3390/nu13062033

**Published:** 2021-06-13

**Authors:** Hee Yun Cha, Soo Jin Yang, Sung-Wan Kim

**Affiliations:** 1 Department of Food and Nutrition, Seoul Women’s University, Seoul 01797, Korea; chaheey@naver.com; 2 Gwang-ju Buk-gu Community Mental Health Center, Gwangju 61261, Korea; 3 Department of Psychiatry, Chonnam National University Medical School, Gwangju 61469, Korea

**Keywords:** dietary inflammation, folate, niacin, schizophrenia, vitamin C

## Abstract

Inflammation is a risk factor for the onset and progression of schizophrenia, and dietary factors are related to chronic inflammation. We investigated whether the dietary inflammatory index (DII) is associated with schizophrenia in the Korean population. Of the 256 subjects who responded to the questionnaire, 184 subjects (117 controls; 67 individuals with schizophrenia) were included in this case-control study. A semi-quantitative food frequency questionnaire was used to evaluate the dietary intakes of the study participants. The energy-adjusted DII (E-DII) was used to assess the inflammatory potential of the participants’ diets. Dietary intakes of vitamin C, niacin, and folate were significantly reduced in the patients with schizophrenia. The patients with schizophrenia had higher E-DII scores than the controls (*p* = 0.011). E-DII was positively associated with schizophrenia (odds ratio = 1.254, *p* = 0.010). The additional analysis confirmed that E-DII was significantly associated with schizophrenia, especially in the third tertile group of E-DII scores (odds ratio = 2.731, *p* = 0.016). Our findings suggest that patients with schizophrenia have more pro-inflammatory diets.

## 1. Introduction

Schizophrenia is a mental disorder that affects approximately 20 million people worldwide [1]. A substantial proportion of patients with schizophrenia do not receive appropriate care due to socioeconomic barriers, especially in low- and middle-income countries [2]. Symptoms of this devastating disease can be classified as positive (hallucinations, delusions, disorganized speech, and disorganized behavior), negative (affective flattening, avolition, diminished interest, and social withdrawal), and cognitive symptoms [3]. Although the etiology of schizophrenia is unclear, studies have found that altering dopamine levels and their functions contributes to the onset of schizophrenia and its pathophysiological changes [4]. Many studies have also identified roles for the dysregulation of other neurotransmitters (e.g., serotonin, glutamate, gamma-aminobutyric acid, and acetylcholine) in the etiology of schizophrenia [5,6].

Anti-psychotic medications acting on dopamine D2 receptor and serotonin (5-hydroxytryptamine, 5-HT) 2A receptor are first-line treatments for patients with schizophrenia [7]. Chronic use of these anti-psychotic medications is often associated with adverse effects on components of metabolic syndrome, including abdominal obesity, atherogenic dyslipidemia (high triglyceride and low high-density lipoprotein cholesterol concentrations), hypertension, insulin resistance, and glucose intolerance. Weight gain, diabetes mellitus, cardiovascular diseases, and metabolic syndrome are also reportedly associated with the use of anti-psychotic medications [8,9]. Patients with schizophrenia require long-term treatment, and many adverse effects of chronic anti-psychotic treatment are related to diet and nutrition. Therefore, a healthy diet and exercise are essential in reducing the incidences of adverse effects of anti-psychotic medications. In [10], patients with schizophrenia had poor dietary habits, including excessive intakes of full-fat creams and carbonated drinks, as well as low intakes of milk and dairy products, vegetables, and fruits. These patients also had lower dietary pattern scores [10].

Inflammation is a protective response against external damage and pathogens. However, chronic or exaggerated inflammatory responses are detrimental to health [11]. Elevated maternal levels of inflammatory cytokines, such as interleukin (IL)-8 and tumor necrosis factor (TNF)-alpha, increase the risk of schizophrenia in the offspring [12,13]. Increased levels of inflammatory cytokines have also been observed in patients with schizophrenia. Studies regarding humans and animals have identified chronic inflammation as a risk factor for schizophrenia [14,15,16,17].

Dietary factors play an important role in regulating the inflammatory response [18,19]. The dietary inflammatory characteristics may signify the presence of chronic inflammation in the body. The dietary inflammatory index (DII) is a population-based index derived from the literature, which was developed to evaluate dietary inflammatory potential. Elevated DII scores have robust validity in indicating an increased inflammatory response, as evidenced by increased inflammatory biomarkers, such as high-sensitivity C-reactive protein (hs-CRP), IL-1 beta, IL-4, IL-6, IL-10, and TNF-alpha [20]. DII has been used to estimate dietary inflammation in various diseases, including colorectal cancer, cardiovascular diseases, and metabolic syndrome [21,22,23,24]. Studies have also been conducted regarding the use of DII in mental illnesses, specifically in patients with depression [25,26]. Few studies have evaluated dietary inflammation in patients with schizophrenia. Two recent studies conducted in Bahrain and the UK showed increased DII and energy-adjusted DII (E-DII) scores in patients with schizophrenia [19,27]. To our knowledge, dietary inflammation in schizophrenia has not been investigated in the Korean population. Understanding the relationship between dietary inflammation and schizophrenia in the Korean population will help to formulate dietary guidelines that can reduce schizophrenia risk and symptom severity.

Here, we investigated the association between dietary inflammation and schizophrenia in the Korean population. The findings of the current study may help to suggest dietary guidelines for schizophrenia subjects, considering the aspect of dietary inflammation.

## 2. Materials and Methods

### 2.1. Study Population and Design

**Figure 1:**
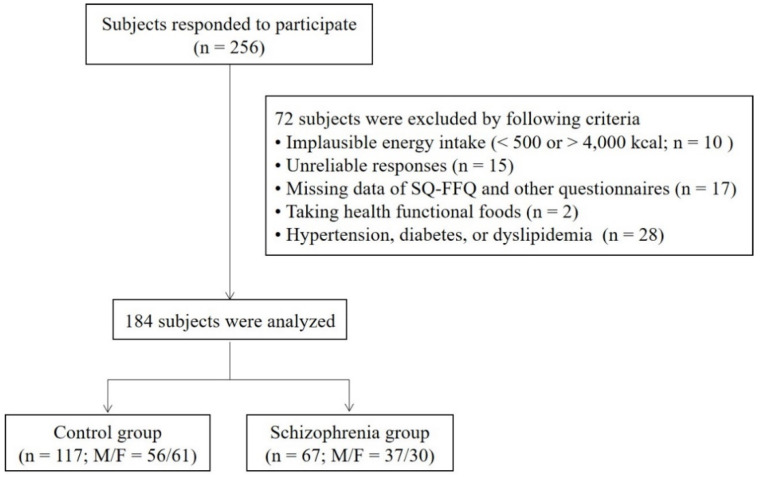
Flow diagram for study participants. SQ-FFQ, semi-quantitative food frequency questionnaire.

The study participants were recruited from the community or community mental health centers in Gwangju, Korea, by posting advertisements. A subset of the registered participants were identical to the cohort discussed in our previous report [28]. We collected data concerning the participants’ general characteristics, including age, sex, presence of metabolic diseases, smoking status, alcohol consumption, changes in body weight over the past six months, and physical activity. Alcohol consumption and smoking status were classified as “yes” if the subject responded that they drink alcohol and smoke every day, respectively. Physical activity was determined by asking about the frequency of moderate exercise (more than 30 min a day). Height and body weight were measured by the examiner. The body mass index (BMI; kg/m^2^) was calculated based on height (m) and body weight (kg). The Asia-Pacific BMI classification was used to categorize participants into underweight (below 18.5 kg/m^2^), normal (18.5 to 22.9 kg/m^2^), overweight (23.0 to 24.9 kg/m^2^), and obese (over 25.0 kg/m^2^) groups. The study questionnaire was completed by 256 people, and 72 subjects (30 control subjects and 42 patients with schizophrenia) were excluded according to the exclusion criteria summarized in Table 1. A total of 184 participants were included in this study (117 controls; 67 individuals with schizophrenia). Participants in the control group did not have any personal or family histories of psychiatric diseases and were not using psychotropic drugs. Schizophrenia was diagnosed in accordance with criteria described in the Diagnostic and Statistical Manual of Mental Disorders, Fifth Edition. All participants were aged 18 to 60 years. None had any current or past histories of hypertension, diabetes mellitus, or dyslipidemia. We excluded participants with serious medical diseases or pregnancy. Based on the responses to the questionnaires, including the semi-quantitative food frequency questionnaire (SQ-FFQ), we also excluded participants with an implausible energy intake (<500 kcal or >4000 kcal), unreliable responses, and those who consumed health functional foods. The flow of study participants is shown in Figure 1. This study was approved by the Institutional Review Board of the Chonnam National University (1040198-150114-HR-003-03). All subjects who participated in this study provided written informed consent. The study was carried out in accordance with the latest version of the Declaration of Helsinki.

### 2.2. Dietary Assessment

The dietary intakes of participants were evaluated using the SQ-FFQ, which had questions concerning 76 food items in the following categories: (1) rice and other grains; (2) fishes and meats; (3) vegetables; (4) oil and sugar; (5) milk and dairy products; (6) fruits; and (7) tea and beverages. The SQ-FFQ included colored photographs of three portion sizes of 17 food items from all food groups. The participants were asked to report the frequencies and portion sizes of 76 food items consumed during the past month. The frequency was reported as never or seldom; once per month; two or three times per month; once or twice per week; three or four times per week; five or six times per week; once per day; twice per day; and three times per day. The portion sizes were classified as small, medium, and large. Computer-Aided Nutritional Analysis Program version 5.0 (Korean Nutrition Society, Seoul, Korea) was used to analyze the dietary intakes of energy and nutrients.

### 2.3. DII Score

In the present study, dietary inflammation was predicted based on the DII calculation previously published [20]. The process of creating the overall DII score for an individual was as follows. We calculated the *z*-score to compare the DII scores of participants in relation to scores from the global database. The z-score was calculated by subtracting the global daily mean nutrient intake from the nutrient intakes of our participants, then dividing the result by the standard deviation (SD) of the mean. A percentile score was calculated for this value to reduce the effect of a “right-skewed” data distribution. The percentile score of each nutrient was converted to a centered percentile through multiplication by 2 and subtraction of 1. Each nutrient’s centered percentile value was multiplied by its overall inflammatory effect score. The DII score of each participant was calculated by summing the DII scores of individual nutrients. To obtain an E-DII score, the dietary intake was converted to intake per 1000 kcal energy intake. A higher DII score indicated a pro-inflammatory dietary potential, while a lower DII score indicated an anti-inflammatory dietary potential.

### 2.4. Statistical Analysis

We analyzed differences in general characteristics, dietary intake, and E-DII scores between participants with and without schizophrenia. The Kolmogorov–Smirnov and Shapiro–Wilk tests were used to assess whether variables were normally distributed. Continuous variables were analyzed using Student’s *t*-test (if the variables were normally distributed) or the Mann–Whitney U test (if the variables were not normally distributed). Categorical variables were analyzed using the chi-squared test or Fisher’s exact test. *P*-values for the statistical differences in dietary intakes between groups were corrected using the analysis of covariance (ANCOVA) model with the nutrients as dependent variables and age, sex, and BMI as covariates. ANCOVA-corrected *p*-value was presented as corrected *P*. Logistic regression analysis was used to investigate the association between E-DII and schizophrenia using an unadjusted model and a model adjusted for age, sex, and BMI. Participants were classified into three groups based on the tertiles (T) of E-DII scores: T1: E-DII < −0.8767; T2: −0.8767 ≤ E-DII < 1.0105; and T3: E-DII ≥  1.0105. The association between E-DII and schizophrenia was analyzed using logistic regression analysis based on the tertiles of E-DII. Statistical analyses were performed using IBM SPSS version 24 (IBM SPSS Statistics, Armonk, NY, USA). Data were expressed as mean ± SD for continuous variables. Statistically significant differences were defined as *p* < 0.05.

## 3. Results

### 3.1. General Characteristics of Study Participants

The general characteristics of the study participants are described in Table 2. Participants with and without schizophrenia had a similar age, sex distribution, height, alcohol use, smoking status, and level of physical activity. However, body weight, BMI, and frequency of obesity were significantly higher in the participants with schizophrenia than in the controls (Table 2).

### 3.2. Comparison of Dietary Intakes between Controls and Patients with Schizophrenia

Total energy intakes were similar between the control and schizophrenia groups (1667.43 ± 607.30 kcal and 1787.42 ± 754.05 kcal, respectively). The energy-adjusted dietary intakes of carbohydrates, proteins, and fats are presented in Table 3. The only statistically significant difference between groups was a reduced dietary intake of arachidonic acid, an n-6 polyunsaturated fatty acid (PUFA), in the schizophrenia group, compared with the control group (corrected *p* = 0.016). The dietary intakes of vitamins and minerals are shown in Table 4. In comparison with participants in the control group, participants in the schizophrenia group had lower dietary intakes of vitamin C (corrected *p* = 0.030), vitamin K (corrected *p* = 0.007), niacin (corrected *p* = 0.003), folate (corrected *p* = 0.042), calcium from vegetables (corrected *p* = 0.021), and phosphorous (corrected *p* = 0.035).

### 3.3. Association between E-DII Score and Schizophrenia

E-DII scores, a measure of the dietary inflammatory potential, were higher in the participants with schizophrenia than in the controls (*p* = 0.011), indicating a pro-inflammatory dietary intake in the patients with schizophrenia (Table 5). Importantly, participants with higher E-DII scores had significantly increased odds of schizophrenia in unadjusted analyses (odds ratio = 1.228, *p* = 0.012) and in analyses adjusted for age, sex, and BMI (odds ratio = 1.254, *p* = 0.010; Table 6). Additional analysis, according to the tertiles of E-DII, also confirmed the positive association between E-DII and schizophrenia, showing an increased odds ratio for schizophrenia in the unadjusted model (odds ratio = 2.471, *p* = 0.019) and in model adjusted for age, sex, and BMI (odds ratio = 2.731, *p* = 0.016), especially in the third tertile group of E-DII, compared with tertile 1 (Table 7).

## 4. Discussion

Our results suggest that pro-inflammatory potentials of diets are associated with schizophrenia. Of the nutrients analyzed, vitamin C, niacin, and folate were especially less consumed in the schizophrenia population. From these findings, encouraging the consumption of anti-inflammatory nutrients may be considered when preparing a dietary guideline for patients with schizophrenia to reduce dietary inflammation and subsequently improve their systemic inflammatory state and related abnormalities.

In comparison with the controls, the participants with schizophrenia in our study had higher body weights, BMIs, and a higher frequency of obesity. The increased body weight and BMI may be attributed to the metabolic effects of anti-psychotic medications [5]. Second-generation anti-psychotic drugs are involved in the pharmacological mechanisms of blocking the dopamine D2 receptor to control psychotic symptoms, and antagonizing the serotonin 5-HT receptor to reduce extrapyramidal side effects liability [5,29,30]. Weight gain and metabolic disorders can occur with the use of second-generation anti-psychotic drugs in patients with schizophrenia. Dopamine is also involved in diet and nutrition. Blocking dopamine D2 receptors on sensory neurons in the lateral hypothalamus increases hunger [31]. When dopamine D2 receptors are blocked, feeding behavior is not properly regulated: this causes overeating and weight gain [32]. In addition, the antagonistic effects of anti-psychotic medications on 5-HT2c, histaminergic (H1), and muscarinic (M3) receptors lead to increased appetite and body weight [33]. Significant increases in BMI have been reported in inpatients with schizophrenia [34]. Female patients with schizophrenia have a higher body fat percentage than do female controls [10]. Abdominal obesity is observed more frequently in patients with a history of multiple episodes of schizophrenia than in controls, as well as in patients with a first episode of schizophrenia [35].

Dietary factors, including omega-3 fatty acids, can affect schizophrenia development and symptom severity [36]. Patients with schizophrenia have increased caloric intakes due to the consumption of high-density foods and low energy expenditures [37]. In addition, patients with schizophrenia have inadequate intakes of several vitamins [28,38]. Supplementation with vitamin B_12_ and folate improves the negative symptoms of schizophrenia [39]. Vitamin D insufficiency and deficiency were observed in patients with schizophrenia [38], and vitamin D intake is negatively associated with the risk of schizophrenia [40]. We previously reported significantly lower dietary intakes of proteins, n-3 PUFAs, vitamin K, vitamin C, niacin, and folate in male patients with schizophrenia, compared with male controls [28]. A previous study found reduced intakes of milk, dairy products, vegetables, and fruits in patients with schizophrenia, compared with the general population [10]. Patients with schizophrenia also had increased intakes of full-fat creams and carbonated drinks, as well as lower dietary habit scores, compared with the general population [10].

Inflammation has been suggested as a potential etiological factor in schizophrenia [41,42]. Increased levels of pro-inflammatory cytokines and microglial activation accelerate schizophrenia’s onset and worsen schizophrenia’s symptom severity [16,17,43,44]. Currently, the most widely accepted hypotheses regarding the etiology of schizophrenia are the “inflammation and two-hit hypothesis” and “inflammation and neural diathesis-stress hypothesis” [45,46]. Excessive inflammation induces the stress response and neurodegeneration, which is a neuronal aging process. Stress response secondary to inflammation sensitizes and primes the microglial cells, thereby increasing the release of pro-inflammatory cytokines. Patients with schizophrenia have higher levels of pro-inflammatory cytokines than do healthy controls [47,48]. In addition, positron emission tomography studies of receptors (PK11195 and DAA1106) have demonstrated that the activation of microglial receptors is associated with neuronal damage in patients with recent-onset schizophrenia [43,49]. Another study showed that higher proportions of microglial cells were bound to the DAA1106 receptor in patients with longer disease duration and worse positive symptoms [50]. Therefore, the evidence thus far implicates inflammation in the pathogenesis of schizophrenia [17,44].

Dietary factors also affect the inflammatory response [18]. Dietary imbalance and dietary inflammation contribute to metabolic alterations related to obesity and metabolic syndrome in patients with schizophrenia. We used the DII to assess dietary inflammatory potential in participants with and without schizophrenia. The DII is a valid indicator of dietary inflammation. Many studies have investigated the associations of DII scores with inflammatory markers. A cross-sectional study (HEalthy Lifestyle in Europe by Nutrition in Adolescents) showed that higher DII scores were associated with increased inflammatory biomarkers, including TNF-alpha, IL-1, IL-2, and interferon-gamma [51]. Similar results were observed in two studies involving the Korean population [52,53]. These studies showed that increased DII scores were associated with higher hs-CRP levels. Numerous studies have shown that the DII is a qualified estimating tool of dietary inflammation, which plays an important role in the systemic inflammatory response. Only two previous studies (from Bahrain and the UK) have evaluated dietary inflammation in patients with schizophrenia [19,27]: dietary inflammation, measured using the DII and E-DII, was significantly increased in patients with schizophrenia in both of these studies. The increased dietary inflammation in patients with schizophrenia in these studies is consistent with the results of our study involving the Korean population. To our knowledge, this study is the first to investigate dietary inflammation using the DII in Korean patients with schizophrenia. As there are no criteria for comparisons between studies, the E-DII scores in the present study cannot be directly compared to other studies. Nevertheless, the data on dietary intake and E-DII scores in the Korean population are significant because they reflect the unique dietary characteristics of the Korean population, with high levels of consumption of various grains, plant foods, and fermented foods [54]. Dietary intakes of vitamin C, niacin, and folate were significantly lower in patients with schizophrenia than in healthy controls. These nutrients have anti-inflammatory properties [20], and their reduced intakes may contribute to the increased dietary inflammation scores in Korean patients with schizophrenia. Our results emphasize the importance of adequate intakes of vitamin C, niacin, and folate in patients with schizophrenia.

Although several studies have been conducted regarding the dietary inflammatory potential in patients with mental disorders and depression [25,26,55], this is the first report of dietary inflammation measured with DII in Korean patients with schizophrenia. Despite its originality, our study had some limitations. First, this study had a modest sample size, and we did not match the cases and controls at a 1:1 ratio. Thus, selection bias may have influenced our results. Second, we could not study the effects of dietary inflammation on the development of schizophrenia because we enrolled patients who had already been diagnosed with schizophrenia. Third, because this was an observational study, we could not identify a causative role for a pro-inflammatory diet in the onset of schizophrenia. Fourth, it is possible that FFQ, the most common dietary assessment tool, under- or over-estimated the individuals’ dietary intakes. Finally, we did not consider psychotic symptoms or anti-psychotics in the present analyses. Further studies, including illness duration, formal scales for psychotic symptoms, or the amounts and types of anti-psychotics, would provide a better understanding of the relation between DII and schizophrenia.

The diets of patients with schizophrenia are generally pro-inflammatory, as evidenced by the higher E-DII scores of the patients with schizophrenia than of the controls. Dietary inflammation was associated with schizophrenia. To reduce dietary inflammation, patients with schizophrenia should be encouraged to maintain adequate dietary intakes of vitamin C, niacin, and folate. These results have implications for developing dietary recommendations for patients with schizophrenia, considering dietary inflammation.

## Figures and Tables

**Table 1:** Inclusion criteria and exclusion criteria for study participants.

	Control	Schizophrenia
Inclusion criteria	Age: 18–60 years	Age: 18–60 years Diagnosed schizophrenia in accordance with criteria described in the Diagnostic and Statistical Manual of Mental Disorders, Fifth Edition
Exclusion criteria	History of psychiatric diseases or usage of psychotropic drugs History of hypertension, diabetes mellitus, or dyslipidemia, or other serious medical diseases Pregnancy Implausible energy intake (<500 kcal or >4000 kcal) Unreliable responses and missing data Consumption of health functional foods	History of hypertension, diabetes mellitus, dyslipidemia, or other serious medical diseases Pregnancy Implausible energy intake (<500 kcal or >4000 kcal) Unreliable responses and missing data Consumption of health functional foods

**Table 2:** Participant characteristics.

	Control	Schizophrenia	*p*
Age (y)	30.17 ± 8.12	32.72 ± 10.78	0.358
Sex (n, %)			0.337
Male	56 (47.9)	37 (55.2)	
Female	61 (52.1)	30 (44.8)	
Height (cm)	167.15 ± 8.52	166.61 ± 9.02	0.697
Body weight (kg)	62.54 ± 14.20	69.26 ± 15.75	0.004
BMI (kg/m^2^)	22.19 ± 3.65	24.97 ± 5.44	0.001
Obesity (n, %)			0.046
Underweight	14 (12.0)	5 (7.5)	
Normal	63 (53.8)	25 (37.3)	
Overweight	15 (12.8)	12 (17.9)	
Obese	25 (21.4)	25 (37.3)	
Alcohol (n, %)			0.059
Yes	7 (6.0)	0 (0)	
Sometimes	20 (17.1)	8 (11.9)	
No	90 (76.9)	59 (88.1)	
Smoking (n, %)			0.445
Yes	17 (14.5)	12 (17.9)	
Sometimes	3 (2.6)	0 (0)	
No	97 (82.9)	55 (82.1)	
Physical activity (n, %)			0.061
Yes	24 (20.5)	22 (32.8)	
Sometimes	37 (31.6)	24 (35.8)	
No	56 (47.9)	21 (31.3)	

Data are expressed as mean ± SD.

**Table 3:** Dietary intakes of energy and energy-adjusted dietary intakes (per 1000 kcal) of carbohydrate, protein, and fats.

	Control	Schizophrenia	*p*	*Corrected P*
Energy (kcal)	1667.43 ± 607.30	1787.42 ± 754.05	0.345	0.442
Carbohydrate (g)	166.98 ± 19.40	172.71 ± 24.98	0.112	0.078
Fiber (g)	13.09 ± 4.56	12.22 ± 6.84	0.020	0.204
Protein (g)	35.76 ± 6.32	33.68 ± 8.07	0.055	0.076
Protein, vegetable (g)	20.31 ± 3.46	19.35 ± 4.67	0.145	0.118
Protein, animal (g)	15.45 ± 6.19	14.15 ± 7.79	0.244	0.252
Fat (g)	21.69 ± 6.55	20.01 ± 8.48	0.167	0.097
Fat, vegetable (g)	10.60 ± 3.68	9.69 ± 4.33	0.131	0.257
Fat, animal (g)	15.45 ± 6.19	14.15 ± 7.79	0.244	0.252
Cholesterol (mg)	154.07 ± 64.13	139.73 ± 73.84	0.173	0.422
Saturated fat (g)	5.06 ± 2.34	5.35 ± 3.66	0.878	0.645
MUFAs (g)	5.26 ± 2.38	5.27 ± 3.35	0.549	0.902
PUFAs (g)	3.44 ± 1.64	3.20 ± 2.10	0.156	0.377
n-6 PUFAs ^1^ (g)	2.94 ± 1.42	2.61 ± 1.68	0.059	0.143
Linoleic acid (g)	2.84 ± 1.40	2.51 ± 1.63	0.050	0.131
AA (g)	0.0081 ± 0.0063	0.0058 ± 0.0064	0.001	0.016
n-3 PUFAs ^2^ (g)	0.44 ± 0.24	0.43 ± 0.33	0.225	0.872
EPA+DHA (g)	0.17 ± 0.14	0.15 ± 0.15	0.108	0.156

Data are expressed as mean ± SD. ^1^ Sum of AA, LA, 20:2(n-6), DGLA, and 22:5(n-6). ^2^ Sum of ALA, DHA, DPA, EPA, and ETA. AA, arachidonic acid; ALA, α-linolenic acid; DGLA, dihomo-γ-linolenic acid; DHA, docosahexaenoic acid; DPA, docosapentaenoic acid; EPA, eicosapentaenoic acid; ETA, eicosatetraenoic acid; LA, linoleic acid; MUFAs, monounsaturated fatty acids; PUFAs, polyunsaturated fatty acids.

**Table 4:** Energy-adjusted dietary intakes (per 1000 kcal) of vitamins and minerals.

	Control	Schizophrenia	*p*	*Corrected P*
Vitamin A (μg RAE)	339.75 ± 180.00	289.10 ± 188.83	0.024	0.161
Vitamin D (μg)	1.62 ± 0.84	1.78 ± 1.28	0.377	0.174
Vitamin E (mg α-TE)	6.16 ± 2.24	5.49 ± 2.24	0.036	0.090
Vitamin K (μg)	114.91 ± 73.51	85.82 ± 68.33	0.002	0.007
Vitamin B_1_ (mg)	0.75 ± 0.18	0.71 ± 0.19	0.037	0.129
Vitamin B_2_ (mg)	0.63 ± 0.19	0.61 ± 0.23	0.503	0.616
Niacin (mg NE)	7.53 ± 1.58	6.77 ± 2.07	0.010	0.003
Vitamin B_5_ (mg)	3.01 ± 0.42	2.98 ± 0.54	0.713	0.990
Vitamin B_6_ (mg)	0.80 ± 0.17	0.74 ± 0.24	0.013	0.077
Vitamin B_7_ (mg)	10.61 ± 5.09	10.07 ± 4.55	0.475	0.956
Folate (μg DFE)	298.24 ± 100.92	264.16 ± 105.29	0.014	0.042
Vitamin B_12_ (μg)	3.45 ± 1.60	3.53 ± 3.18	0.222	0.526
Vitamin C (mg)	59.76 ± 28.91	49.37 ± 31.38	0.008	0.030
Calcium (mg)	261.47 ± 93.46	245.19 ± 122.45	0.089	0.436
Calcium, vegetable (mg)	137.17 ± 56.38	117.31 ± 60.45	0.013	0.021
Calcium, animal (mg)	122.21 ± 63.91	112.90 ± 76.77	0.207	0.789
Chloride (mg)	172.66 ± 102.63	167.06 ± 115.22	0.628	0.870
Iron (mg)	7.12 ± 1.47	6.61 ± 1.89	0.011	0.066
Iron, vegetable (mg)	5.56 ± 1.35	5.21 ± 1.61	0.122	0.109
Iron, animal (mg)	1.56 ± 0.65	1.40 ± 0.74	0.055	0.257
Magnesium (mg)	41.71 ± 19.91	42.96 ± 27.96	0.626	0.672
Phosphorous (mg)	569.24 ± 102.46	532.23 ± 130.37	0.048	0.035
Potassium (mg)	1557.45 ± 451.39	1448.75 ± 525.94	0.085	0.144
Selenium (μg)	51.00 ± 9.20	49.23 ± 11.50	0.283	0.357
Sodium (mg)	1815.54 ± 707.05	1809.80 ± 943.05	0.582	0.833
Zinc (mg)	5.43 ± 0.85	5.13 ± 0.96	0.033	0.061

Data are expressed as mean ± SD. DFE, dietary folate equivalents; NE, niacin equivalents; RAE, retinol activity equivalents; TE, tocopherol equivalents.

**Table 5:** Energy-adjusted dietary inflammatory index (DII) scores of control and schizophrenia subjects.

	Control	Schizophrenia	*p*
Energy-adjusted DII	−0.25 ± 1.91	0.56 ± 2.13	0.011

Data are expressed as mean ± SD.

**Table 6:** Odds ratios (95% confidence intervals) for schizophrenia by energy-adjusted dietary inflammatory index (DII) scores.

	Model 1	Model 2
Energy-adjusted DII scores	1.228 (1.046–1.441) *p* = 0.012	1.254 (1.055–1.490) *p* = 0.010

Associations were explored by logistic regression analysis. Model 1: unadjusted; Model 2: adjusted for age (1-year increment), sex, and body mass index (1 kg/m^2^ increment).

**Table 7:** Odds ratios (95% confidence intervals) for schizophrenia according to the tertiles of energy-adjusted dietary inflammatory index (E-DII) scores.

Energy-Adjusted DII Scores	Tertile 1	Tertile 2	Tertile 3
Model 1	Reference	0.824 (0.366–1.854) *p* = 0.639	2.471 (1.159–5.269) *p* = 0.019
Model 2	Reference	0.868 (0.369–2.043) *p* = 0.746	2.731 (1.210–6.165) *p* = 0.016

Associations were explored by logistic regression analysis. Model 1: unadjusted; Model 2: adjusted for age, sex, and body mass index. Tertile ranges of energy-adjusted DII: T1 (E-DII < −0.8767), T2 (−0.8767  ≤  E-DII < 1.0105), and T3 (E-DII ≥1.0105).

## Data Availability

The data presented in this study are available upon reasonable request to the corresponding author.
